# Starstruck by journal prestige and citation counts? On students’ bias and perceptions of trustworthiness according to clues in publication references

**DOI:** 10.1007/s11192-022-04521-4

**Published:** 2022-10-10

**Authors:** Evelyn Eika, Frode Eika Sandnes

**Affiliations:** Department of Computer Science, Faculty of Technology, Art and Design, Oslo Metropolitan University, St OlavsPlass, P.O. Box 4, 0130 Oslo, Oslo Norway

**Keywords:** Trust, Credibility, Post truths, Publication, References, Citations, I21, I23, I29

## Abstract

Research is becoming increasingly accessible to the public via open access publications, researchers’ social media postings, outreach activities, and popular disseminations. A healthy research discourse is typified by debates, disagreements, and diverging views. Consequently, readers may rely on the information available, such as publication reference attributes and bibliometric markers, to resolve conflicts. Yet, critical voices have warned about the uncritical and one-sided use of such information to assess research. In this study we wanted to get insight into how individuals without research training place trust in research based on clues present in publication references. A questionnaire was designed to probe respondents’ perceptions of six publication attributes. A total of 148 students responded to the questionnaire of which 118 were undergraduate students (with limited experience and knowledge of research) and 27 were graduate students (with some knowledge and experience of research). The results showed that the respondents were mostly influenced by the number of citations and the recency of publication, while author names, publication type, and publication origin were less influential. There were few differences between undergraduate and graduate students, with the exception that undergraduate students more strongly favoured publications with multiple authors over publications with single authors. We discuss possible implications for teachers that incorporate research articles in their curriculum.

## Introduction

Several initiatives aim to make research more accessible to the public. For example, Plan S was established to ensure that both publicly and privately funded research are published in open access channels (Kiley & Smits, 2019). Open access publications can be read by anyone without institutional access to digital libraries behind payment walls. Many researchers also connect with society by sharing and discussing their research using social media, blogs, and videos. Institutional initiatives to disseminate research via traditional media channels, such as newspapers, have become more intensified and professionalised over the years. A research discourse is characterised by debates, discussions, disagreements, and diverging views (Barzilai et al., 2015; Sadler, 2004; Strømsø, 2013). Readers may therefore be faced with controversial and diverging claims which they have to resolve.

Certain stakeholders may be tempted to abuse research to divert public opinion and mislead policy makers through “fake news” (Herrick, 2001; Wen et al., 2020; Zhou & Zafarani, 2018). “Fake news” are often connected with socio-scientific issues, for example, effects of vaccinations, health risks of cell phones and tobacco, power transmission lines and cancer, cloning, genome projects, stem cells, climate change, pollution, etc. Misinformation about the COVID-19 pandemic has been coined an “infodemic” (Zarocostas, 2020). “Post-truth” is another term that refers to misinformation or science denial (Barzilai & Chinn, 2020; Damico et al., 2018).

Several scholars argue for lateral reading strategies when evaluating claims (Breakstone et al., 2018; Wineburg & McGrew, 2019). Still, the literature on science education seems to suggest that untrained readers tend to use a range of different strategies and available information when evaluating claims (Bråten et al., 2009). Although some studies have addressed effects of reference information such as publication type, publisher, and publication date (Bråten et al., 2009; Kolstø et al., 2006), this avenue of enquiry appears comparatively less explored than other factors. Even though references may play a small role in the overall assessment, there may be a risk that such evaluations become biased due to stereotypical perceptions or a lack of knowledge. With the backdrop of publicly available research discourse, an assumed public interest in research, and the emerging accessibility and use of bibliometric markers via the web, the objective of this study was to observe to what degree untrained readers’ perceptions are biased by publication information.

The ability to vet research publications is a skill that develops with experience and training. After successfully completing doctoral training, researchers are expected to understand the research system in general and understand the publication practices and traditions specific to their own academic field in detail. Yet, the assumption that trained researchers are highly knowledgeable about research assessment may not always hold. For instance, Kamrani et al. (2021) observed that about 40% of the researchers questioned had an incorrect understanding of the h-index.

Higher education administrators comprise another group of individuals who may not have research training yet who may make decisions based on publication reference information in the context of recruitment, promotion, resource allocation, and research management (Haugen & Sandnes, 2016; Sandnes, 2018). Clearly, such decisions can have severe implications. Misinformation and conspiracy theories can impair individuals’ abilities to make sensible decisions. Such decisions can affect their health, personal finances, and other key aspects of their lives, as well as voting in elections (Covitt et al., 2013). This study intended to gain insight into how non-experts are biased by reference information. These non-experts were represented by students that are not explicitly pursuing a research career.

### Does reference information trigger bias?

A reference typically includes standardised information (e.g., APA, MLA, ISO 690, etc.) such as the name of the authors and the type and year of publication. It is also relatively easy, with a little bit of extra effort, to determine how many times a work is cited using a database such as Google Scholar (Sandnes, 2020). Some researchers may weigh publications according to the impact factor of the journal where the publication appears or according to the prestige of where the journal is indexed. We did not include such factors in this study as it was assumed that most untrained readers are unfamiliar with impact factors and indexing and where to locate such information.

Much of the literature on research assessment relates to citations and various indicators derived from citation counts (Lindsey, 1989; Onodera & Yoshikane, 2015; Phelan, 1999). Citations accumulate over time and are therefore not provided in a traditional printed publication. One exception is papers that specifically address citations and thus may include a static snapshot of the citation counts at the time of writing. Still, citation information is easily retrievable with publicly available tools such a Google Scholar although citation statistics differ across databases (García-Pérez, 2010; Gehanno et al., 2013). Citation numbers can also be misleading if the citations are negative (Xu et al., 2022), i.e., reflect a criticism of a specific work, if citations are related to non-scientific aspects of the publication (Mammola et al., 2022; Tahamtan & Bornmann, 2019), promoted as a journal cover paper (Rachatasumrit et al., 2022), or if the citation totals contain many self-citations (González-Sala et al., 2019). It is thus possible that a problematic work accumulates a high citation count based on critical comments, or an unimportant work gains a high citation count because of a researcher’s self-promotion strategy to gain visibility. Although citation counts can represent criticisms, such citation counts still indicate that the contribution was a relevant part of the research discourse. Important research breakthroughs may sometimes result from what has been learned by previous failed attempts of other researchers. Lyu et al. (2021) provided a useful overview of the underlying motivations to cite. The availability of citation indicators does not mean that readers will utilize such information correctly. In fact, Petrovich (2022) observed that bibliometric indicators influenced the formation of facts in the Italian press, and that the voices of experts on bibliometric indicators are comparatively underrepresented. Consequently, Petrovich argued that objective knowledge about bibliometric indicators does not adequately reach the general public. Clearly, considering citation counts superficially without context and expertise can be problematic. Although citation counts could give readers the impression that a work is trustworthy, such connections between impact and trustworthiness are not justified. It was thus relevant to observe if untrained readers place similar emphasis on citation counts as some researchers do, namely:

#### H1

Untrained readers perceive references to papers with many citations more trustworthy than papers with fewer citations.

The publication date, typically the year, is nearly always present in a reference record. With reference standards such as APA it is also visible in the in-text citations. Clearly, the publication date can serve as a proxy for how timely or leading a research work is (Glänzel & Schoepflin, 1995; Hörlesberger et al., 2013; Klavans & Boyack, 2008). A recently published work may be considered more current and perhaps more relevant in the current context than a work published many years ago. Bråten et al. (2011) reported that their cohort of students placed more emphasis on actual content than date of publication when assessing the trustworthiness of documents. In technological fields with rapid paradigm shifts, time of publication may play a more prominent role than in fields that change more slowly. We thus formulated a hypothesis to test if students exhibit similar emphasis on recency of publication as some researchers, namely:

#### H2

Untrained readers perceive references to recently published work more trustworthy than less recently published work.

The author list is a noticeable attribute of a reference. The author list is usually the first entry in a reference record and the first information the reader encounters. Moreover, reference standards such as APA also include author information in the in-text citation. The author list in a reference provides several clues. The most important is perhaps the number of authors, that is, it is a single authored publication, or it is a collaboration between several authors. Sceptics may argue that publications authored by multiple authors may acquire higher citation counts due to self-citations by the individual authors and not necessarily because of quality. However, it may also seem intuitive, especially for students who have first-hand experience with sociocultural learning and teamwork, that research resulting from collaborations that draw on varied expertise of a team would be of higher quality and consequently become more frequently cited than lone-wolf research. Indeed, the results of several studies support this view (Bartneck & Hu, 2010; Bu et al., 2018a, 2018b; Larivière et al., 2015; Melin & Persson, 1996; Sandnes, 2021a; Wuchty et al., 2007; Xu et al., 2015). Contrastively Aksnes and Aagaard (2021) pointed out that “there is a common perception of highly cited researchers as individual geniuses who can be singled out for their extraordinary contributions”. This dichotomy gave rise to the following hypothesis.

#### H3

Untrained readers perceive references with multiple authors more trustworthy than single authored references.

To determine if students resolve conflicts according to the formal expertise of the source, Thomm and Bromme (2016) did a controlled experiment where they divided the students into four groups and presented a pair of conflicting claims which were made by a university professor versus a professor from another university (control), a junior researcher, or a researcher in industry. Responses from a six-item Likert type questionnaire probing their subjective explanations revealed that the respondents endorsed the claims of the professor over the junior researcher. No differences were observed between the two university professors. When the conflict was between the researcher in industry and university professor, the respondents were more likely to agree more with explanations related to their personal motivation.

An author may also be a well-known researcher, for example, a Nobel prize winner. One would expect that readers trust works by researchers they know of. However, a beginner-level researcher may be less familiar with the celebrities of a scientific field and therefore impartial to the authors’ reputation. Also, it is challenging to quantify how well-known an author may be. We thus decided not to explore fame. We have also not included how readers consider references to well-known organisations such as the World Health Organisations and the United Nations that publish highly cited statistics.

Another clue in the author list is the origin of the author implied by the spelling of the name, for example, common names in English speaking countries, German names, Chinese names, Indian names, etc. There are no valid arguments to suggest that the name should have any effect on the quality of research especially as research talents can be found all over the world, and academics make up a highly internationally mobile profession (Velema, 2012). In fact, Long et al., (2009) found that researchers are not biased by the origin of a researcher but rather where the researcher received their education as pedigree is valued among academics. The assessments by untrained readers may be affected by prejudice and thereby unconsciously or consciously favour research conducted by authors with a name rooted in high GDP-per-capita countries compared to low GDP-per-capita countries. GDP-per-capita is sometimes used as a geo-economic characteristic of countries in scientometric studies (Hart & Sommerfeld, 1998; Vinkler, 2008; Ye, 2007). We therefore formulated a hypothesis to test if untrained readers are biased by stereotype perceptions of authors’ geographic origins, namely:

#### H4

Untrained readers perceive references by authors with names associated with high GDP-per-capita countries as more trustworthy than authors with names associated with low GDP-per-capita countries.

An attribute that may be assessed without specific knowledge is the name of the publication. Some journals and conferences carry names that relate to a geographical region. International publication channels are typically considered superior to national publication channels (Meneghini et al., 2006; Nazarovets, 2020; Rabinovich, 1992). For example, the CORE (Computing Research and Education) and CCF (China Computing Federation) conference rankings only list international conference venues (Li et al., 2018). We wanted to explore if respondents were biased regarding publication channel names when contrasting publication channels containing country names associated with high versus low GDP-per-capita. Hence the following hypothesis was formulated:

#### H5

Untrained readers perceive references to papers published in publication channels hosted in high GDP-per-capita countries more trustworthy than papers in publication channels hosted in low GDP-per-capita countries.

A publication channel is usually connected to a publisher, and the publisher can also provide valuable clues. A trained researcher is more likely to place trust in a known publication channel by a publisher with a prestigious reputation compared to an unknown or less reputable publisher. A publication channel may also carry the name of a different country than the country of the publisher, for example, an “American journal” published in Switzerland. To complicate things further, some journals are sponsored by special interest organisations, with varying levels of prestige. Such an organisation may be labelled as international, yet the publications may be carried by a local publisher with a national distribution scope. The title “international” is not protected by regulations and can thus be used by anyone. Untrained readers are unlikely to be able to distinguish reliably between the reputation of publishers and organisations. These dimensions were not included in this study.

The publication channel in which a work is published is usually provided in a reference. Experienced researchers are familiar with the reputation and quality of the main publication channels within their respective fields. However, we were interested in how inexperienced researchers relate to publication channel information. In fact, Bråten et al. (2011) found that undergraduate students trusted textbooks and official documents more than newspapers. They also found that students with low knowledge about a topic were more likely to trust less trustworthy documents. Kobayashi (2014) found that students tended to place more trust in claims supported by credible sources. Although able to use source credibility to resolve conflicts, Kobayashi concluded that the students were not good at this process. Also, individuals’ perceptions of how credible a publication channel is can be incorrect. As commented by Breakstone et al. (2018), Wikipedia has a somewhat low reputation as an information source since it can be edited by anyone, at least in theory. Breakstone et al. argued that Wikipedia can be a suitable starting point for students conducting research since entries generally are carefully reviewed and that only a small number of individuals can edit high-traffic Wikipedia entries. With the cohort of respondents in this study (technology students), it was relevant to address conference proceedings versus journal papers. In most scientific fields, journal papers are considered more archival and enjoy a higher academic status than conference papers. Still, this dichotomy is less relevant in fields where results are predominantly published as monographs. Also, it may increasingly be less important in certain fields such as areas within computer science where quality conferences are valued on par with prestigious journals (Franceschet, 2010; Kim, 2019; Lee, 2019; Mannocci et al., 2019; Sandnes, 2021a). We thus formulated the following hypothesis:

#### H6

Untrained readers perceive references to journal papers more trustworthy than papers in conference proceedings.

This hypothesis assumes that the readers are knowledgeable about the differences between conference proceedings and journals.

References traditionally have included page numbers. One could hypothesise that readers may find a publication with many pages more trustworthy than a publication with fewer pages. Often work in progress may be published in shorter formats and later followed up with longer, more complete and elaborate archival works (Mubin et al., 2018). However, the number of pages is not a reliable measure of publication length as the number of words per page depends on the typographic layout. The layout varies from dense two column A4 pages and others to sparse A5 single column formats with large margins. Moreover, a short text may provide more substantial and relevant contributions than a long unfocused and wordy text. Further, with recent online publications it is becoming less common to provide running page numbers with the publications. A purely web-oriented text is not divided into pages. We thus decided not to include page numbers in this study.

The online interest in papers has been found to correlate with abstract readability (Jin et al., 2021). Recent developments such as video summaries have also been shown to be effective in helping research studies gain traction (Zong et al., 2019). In this work we discard important information that is found within the paper itself or on the web page of a publication such as the abstract, authors affiliations, bio, video summaries, keywords, funding information, references, and the actual contents, as this assumes that the reader is doing a more thorough and deep review of the work.

The rest of this paper is organised as follows. The next section gives an overview of research into science literacy that is related to this study. Next, the details of the methodological approach are provided. This is followed by a presentation of the results. The results are then discussed, and implications are outlined. The paper closes with concluding remarks.

### Related work

In the information-rich society individuals need certain competences, such as to be able to distinguish sponsored contents from non-sponsored contents, advertisements from regular contents, propaganda from dispassionate analysis, opinion pieces from factual pieces, and assess the reliability of information on websites (McGrew et al., 2018). Social media platforms present several challenges such as algorithm bias in how and what news are aggregated, filter bubbles, opinion echo chambers, unwillingness to express minority opinions due to spirals of silence, misconceptions, biases due to false consensus effects, and intentional disinformation (Höttecke & Allchin, 2020).

How laypersons and students perceive and trust science claims is a key issue that has received much attention within the domain of science education (Sadler, 2004). Barzilai and Chinn (2020) presented a framework with four categories that are useful in understanding underlying reasons for the prominence of post truths, namely, *not knowing how to know*, *fallible ways of knowing*, *not caring about the truth,* and *disagreeing about how to know*.

*Not knowing how to know* can refer to situations where individuals lack the knowledge about how to check facts and claims and detect media bias. Consequently, many science educators focus on approaches for strengthening students’ media and science literacies from an early age (Cooper, 2011; Covitt et al., 2013; Kolstø, 2001b).

*Fallible ways of knowing* refers to individuals’ limited cognitive abilities to evaluate claims. For example, individuals may be misled by illusionary effects, or be subject to confirmation bias, i.e., the phenomenon where we tend to place more trust in information that is repeated (Foster et al., 2012). Another phenomenon is where individuals prefer intuitive, quick, and automatic thinking over deeper reflections (Cooper, 2011; Covitt et al., 2013; Kahneman, 2011). The promotion of epistemic vigilance is suggested as one approach for overcoming fallible ways of knowing. In short, epistemic vigilance involves taking into consideration the risk of being misinformed and considering both sides of an argument when evaluating claims.

*Not caring about the “truth”* refers to a lack of motivation to seek insight and understanding. A lack of motivation could be due to the feeling of powerlessness with an Internet populated with misinformation. Barzilai and Chinn (2020) argued that this motivation may be reduced in a climate where politicians are not penalized for presenting misinformation. One remedy they suggest is for scholars to emphasize their position as role models and establish enthusiasm for seeking insights and understanding.

*Disagreeing on how to know* refers to situations where individuals hold values where certain ways of knowing are superior to others thereby brushing off claims. For example, findings that challenge someone’s political ideology are likely perceived as more uncertain than findings that do not (Broomell & Kane, 2017). One remedy to prevent such situations is to train students in discussing disagreements and to reflect over other points of view.

Much of the research on readers’ information and science literacy have addressed student’s sourcing strategies and trustworthiness of online information (Pickard et al., 2010; Shah et al., 2015). Sourcing can be understood as the process of identifying the source of a document, referencing documents, or using source information to evaluate a document’s content or its trustworthiness. Breakstone et al. (2018) suggested that students should be taught to engage in lateral reading (study other information that gives clues to the trustworthiness of a source) before vertical reading (reading the actual text). In a study of fact-finding experts’ best practices on the Internet, Wineburg and McGrew (2019) found that the expert fact checkers read laterally, leaving websites after a quick glance to inspect the website credibility, while the non-expert fact checkers tended to read vertically and were easily misled by official-looking logos and domain names. The rhetorical style of a textbook may lead readers to blind trust (Paxton, 2002). Stadtler and Bromme (2007) found that students with low topic knowledge used unsuitable criteria for assessing websites such as first impressions and web page layout. Brand‐Gruwel et al. (2017) observed that domain experts exhibited more developed use of evaluation criteria and found more reliable information on the web. Similarly, individuals with a science background were observed to value empirical consistency to a greater degree than individuals without a science background when connecting arguments and conclusions (Hogan & Maglienti, 2001).

Many of the studies have focused on non-experts. For example, in a study involving 122 students, Bråten et al. (2009) found that readers use a variety of available information when assessing the trustworthiness of sources, but that most emphasis was placed on the contents compared to document markers. Of the document markers, they found that the respondents placed more emphasis on document type and publisher than publication date and authors. Similarly, Barzilai et al. (2015) also found that novices employ a range of sourcing practices.

Observations of university students asked to evaluate conclusions in news briefs (Korpan et al., 1997) showed that the students asked for quite varying types of information. Most asked for details about the methodology and why the results occurred, while fewer students asked for information about the findings, the people behind the studies, and where the studies were conducted. Least requested were information about related research. A study of printed media (Zimmerman et al., 2001) found in newsagents reporting science results showed that these rarely include information requested by students (theory, method, and data) or recommended by experts (related work and relevance).

Kolstø (2001b) classified how 16-year-olds approached a socio-scientific issue into four resolution strategies: 1) acceptance of knowledge claim, 2) using reliability indicators to evaluate claims, 3) acceptance of authoritative sources, and 4) evaluation of sources. Paul et al. (2017) set out to explore the gap between 9th graders’ knowledge about sources and the degree of which they use source information. Their interviews confirmed that the students have knowledge about sourcing. The data revealed that the underlying reason the students did not make use of this knowledge was mainly a lack of motivation to do so and their focus on content. A study of source selection strategies (List et al., 2016) showed that students justified their selections more according to relevance and accessibility (non-epistemic) than reliability and credibility (epistemic).

Successful sourcing strategies involve using the Internet, forming search queries, and handling search results. A study by Salmerón et al. (2013) confirmed the top-link heuristic whereupon they tend to choose the top first results from a search engine. However, the respondents engaged in more deliberate considerations when bookmarking links to pages.

How readers rely on their own knowledge when analysing opposing views has been addressed by several scholars. For instance, Strømsø et al. (2013) observed that their respondents generally relied on their own knowledge when analysing an issue from multiple angles rather than the sources. In an interview study on how students with varying backgrounds considered arguments on climate change, Damico et al. (2018) found that the respondents solicited the opposing arguments and more evidence in support of claims. They also observed that respondents tended to lean on their knowledge, experiences, and personal perspectives, and concluded that this trend is problematic as readers are inclined to trust arguments that fit with their pre-existing beliefs. Using read aloud tasks involving online conflicting documents, Anmarkrud et al. (2014) found that respondents placed more trust in unbiased sources (scientific texts based on empiric evidence) and less trust in biased sources (subjective newspaper articles).

In addition to trustworthiness, scholars have also studied how students handle uncertainty of results (Guillaume et al., 2017; Metz, 2004) and tentativeness of results (Kimmerle et al., 2015) communicated in science. Covitt and Anderson (2022) argued that students should learn to understand uncertainty in their own data and seek to understand uncertainty analyses in others’ work. Interviews conducted by Schroeder et al. (2019) indicated that schoolchildren recognized uncertainty in personal science (their own activities), and that exposure to uncertainty in personal science led to an understanding of uncertainty in formal science (scientists’ activities). They therefore suggested that schoolchildren should explore and learn through personal science activities. Based on a study involving more than 300 undergraduate students, Lombardi et al. (2014) found that plausibility was predicted by the perceived certainty of claims and source credibility.

Kimmerle et al. (2015) concluded that journalists reporting on medical research news should constantly remind readers about the tentativeness of results in that knowledge is not absolute – it is a dynamic entity that is often revisited and revised. As reflected by Feinstein & Waddington (2020) science does not provide absolute truths about the world, but rather “good enough” fragments, or tools, that help us act in the world. It was suggested by Sinatra and Lombardi (2020) that source evaluation should be combined with plausibility assessments of claims and how well these are connected to data.

The methods for teaching science literacy have also received attention. For example, Wiley et al.’s (2009) controlled study of students’ internet sources evaluation and use showed a positive effect of instruction. A positive correlation was observed between learning outcomes and the ability to distinguish between reliable and unreliable information. The instructions included identifying the authors, their expertise, and motivation, whether the information is based on scientific results, corroboration of information across multiple sources, and whether the information makes sense and fits their existing understanding. Feinstein and Waddington (2020) argued for the sociocultural perspective where students collectively relate to science in social contexts drawing on the diversity of the team members, thereby overcoming limitations of individual judgements. In a study of how education students examined scientific texts in teams, Kolstø et al. (2006) found that about a quarter of the student groups considered the quality of references with arguments such as “relevant references”, “prominent scientific journals”, or “trustworthy and respected sources”. Nearly half of the student groups considered the consistency of arguments and the face validity of arguments. The groups also used criteria related to the completeness of information such as completeness of references and arguments and the one-sidedness of arguments. Criteria related to social aspects of the sources were also considered such as underlying interests, personal values, author competences, professional recognition, and level of expert agreement.

## Method

A review of the literature gave rise to the six hypotheses stated in previous sections. These hypotheses predict how laypersons perceive trustworthiness of information about citation counts, publication date, number of authors, author name, publication channel origin, and publication channel type in scientific references. To support or refute these hypotheses we decided to use a closed-question questionnaire as these can be practical and effective in acquiring the opinions of many individuals. The following sections detail how the questionnaire was designed, from whom and how the responses were solicited, and how the data were analysed.

### Questionnaire design

A three-part questionnaire was designed. The first part of the questionnaire probed how respondents spontaneously vet references without explicit instructions of what feature to rely on. These questions thus attempt to capture how respondents respond in practice. This part comprised 18 questions with a set of contrasting pairs of fictitious claims connected to corresponding fictitious references. The following example probed if date of publication affects a respondent’s trust:

Chen (1968) found that the treatment did not slow cancer, while Wang (2018) found that the treatment was very effective in slowing cancer. Do you trust Chen or Wang?

Chen (1968). Journal of Cancer Studies (cited 23 times).

Wang (2018). Journal of Cancer Research (cited 23 times).

Each pair of contrasting references were provided on the opposite sides of a 5-item Likert scale. A 5-item Likert scale was chosen as it can capture nuances in certainty, while not being too daunting. Respondents were instructed to choose between the pairs of respective claims using the reference information. The references were designed such that only the factor in question was varied. This approach is similar to the methodology used by Thomm and Bromme (2016) to assess perceptions of scholarly expertise.

The 18 questions were designed to examine six reference qualities corresponding to the six hypotheses, namely, the number of citations, the publication date, the number of authors, the origin of the authors based on name, the origin of the publication channel according to its name, and the type of publication. To ensure more reliable sampling of responses, three different instances of each of the six attributes were created.

The second part of the questionnaire probed the participants’ ranking of the six reference features. This part comprised 15 questions contrasting each feature pair corresponding to all the combinations of the six features. For example, the author names and publication date were contrasted using the following:

I trust a paper according to

the names of the authors.

how recently the paper was published.

Note that the terms such as author and recently were not explicitly defined. Each pair of contrasting features were provided on the opposite sides of a 5-item Likert scale. Unlike the part of the questionnaire that measured what respondents would do in practice, this part probed what respondents said they would do in theory.

The questionnaire also solicited general information in the third section about the participants’ experience with research, their self-assessed knowledge about research, and their aspiration with regard to learning more about research. A free-text field for commenting was provided at the end of the questionnaire.

The presentation order of the questions within each part was randomised. Moreover, the direction of Likert dimensions for the individual questions was also randomised. All respondents received the questions in the same order.

Two versions of the questionnaire were designed, one in English and one in Norwegian, to be used in the classes taught in English with international students and Norwegian with local students, respectively.

### Procedure

The questionnaires were implemented using Google Forms. The questionnaire was administered during a period of two years as part of different courses related to research. The cohorts were therefore closely controlled. The invitations to participate were distributed electronically in the learning management system between classes. The results for each specific class were shared and discussed with the respondents afterwards. Hence the questionnaire was also used as a pedagogical tool to increase the students’ knowledge about and reflection on research and publications.

This study was conducted according to national regulations and institutional guidelines. Participation was anonymous and voluntary. As no personal or sensitive information was collected, data handling approval procedures did not apply (Sandnes, 2021b).

### Analysis

As the direction of the dimensions of the Likert scale questions in the questionnaire was randomised, the direction of the responses was first converted into a unified direction. For the first part of the questionnaire the median responses to each of the six types of questions were taken (three responses per question type).

Next, the aggregated results were ranked for each of the three parts of the questionnaire according to their offset from the centre of the scale. First, the responses were mirrored to the same side of the scale using

$$b = \frac{{L_{1} + L_{2} }}{{L_{4} + L_{5} }}$$

where *L*_*1*_ is the number of 1 Likert responses, *L*_*2*_ is the number of 2 responses, etc. That is, if *b* was less than 1, the direction of the scale was mirrored. The final rank order was given by *b* of the mirrored questions.

The data were analysed using JASP 0.16.0.0 (JASP, 2021). Non-parametric tests were used due to the ordinal nature of the Likert responses. Diverging stacked bar graphs (Heiberger & Robbins, 2014) were used to visualise the responses as these clearly reveal the trends of the responses and their certainty, that is, whether the responses are biased towards either side of the scale. Moreover, these plots reveal the distribution of the responses.

### Participants

This study follows the practices of science education research by targeting students (Barzilai & Chinn, 2020; Sadler, 2004). Students were considered not to be too deeply indoctrinated in the research traditions and their values, but with sufficient insight to recognize the problems raised and reflect over these dilemmas. This cohort will be referred to as *untrained readers* herein as they have not completed formal research training. Master students usually receive some basic introduction to research, and they are commonly expected to incorporate a literature review within their master theses. Only a very small fraction of master students pursue a research career. PhD studies train students for the research profession, and doctoral students are often expected to publish. Some master students, and even bachelor students, also have their work published. Through practising writing literature reviews, students become more aware of the components of the research articles, including citations, references, and timeliness (Eika, 2021). Traditionally, bachelor students received little training in research. Yet there has been an increasing focus on research-based teaching and the involvement of undergraduates in research (Healey et al., 2010). It would be challenging to target such a study towards the general population as most citizens may have a limited interest in research, may have never written an academic text, and may thus be unfamiliar with this type of dilemmas.

A total of 148 students at bachelor, master, and PhD levels were recruited for this study over a period of two years. Students were invited from several classes at the three respective levels. Participation was voluntary and the response rate was in the range of about 10% to 20%. All the students recruited studied technology at Oslo Metropolitan University in Norway, of which a majority studied information technologies. Three incomplete responses were discarded resulting in 118 bachelor (undergraduate) student responses, 20 master student responses, and 7 PhD student responses. The master and PhD student responses were combined into 27 graduate level responses. We will use the North American nomenclature and refer to the master and PhD students collectively as *graduate students* herein.

We did not probe the participants’ gender as it was not considered an influential factor. Nor did we probe the participants’ age. However, it is assumed that on an aggregated level the bachelor students would be the youngest group and PhD students the oldest group.

The students recruited were enrolled in several courses that in some way addressed the research process which provided a context to the questionnaire. Several questions were asked to establish a baseline of the respondents’ familiarity with research. Among the graduate students 96.2% reported they had read a research paper, while 90.7% of the undergraduate students reported having read a research paper. More than half of the graduate students reported having some research knowledge, while the self-reported knowledge about research among the undergraduate students was more varied. A Mann–Whitney test shows that the difference in self-reported knowledge about research was statistically significant (*W* = 2182.5, *p* = 0.002, *ES* = 0.370). Note that the rank-biserial correlation effect size (ES) is reported herein.

We also probed if the participants had first-hand experience with research. Among the graduate students 51.9% responded that they had been involved in the writing of a research paper, while 20.3% of the bachelor students reported having been involved in the writing of a research paper.

## Results

### Effect of reference clues

**Fig. 1:**
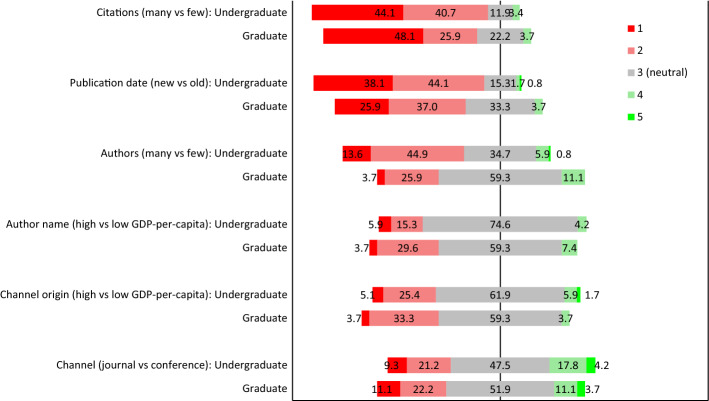
A diverging stacked bar graph showing the percentage distribution of respondents’ perceived trust in six publication reference attributes on a 5-item Likert scale

Figure 1 shows a summary of the results relating to how the respondents were implicitly influenced by reference attributes. The number of citations was clearly associated with the largest offset from neutral as most of the respondents found references with many citations more trustworthy than references with few citations.

Date of publication was the second most influential factor as most respondents placed more trust in a recent reference when contrasted against a more dated reference.

The number of authors was the third most influential factor. However, undergraduate students expressed a stronger trust in publications with multiple authors compared to single authored publications than the graduate students whose responses were dominated by neutral responses. A Mann–Whitney U test shows that this difference was statistically significant (*W* = 2078.0, *p* = 0.008, *ES* = 0.304). This difference between undergraduate and graduate students was also the only one to be statistically significant among the six reference attributes.

Regarding the two factors related to the origin of the author and the publication, that is, high vs low GDP-per-capita countries, the results revealed a small bias towards more trust in authors with a name from high GDP-per-capita countries and more trust in publications channels hosted in high GDP-per-capita countries. The bias was somewhat larger for graduate students compared to undergraduate students although this difference was not statistically significant (author name origin *W* = 1466.5, *p* = 0.418, *ES* = 0.079, and publication origin *W* = 1464.5, *p* = 0.453, *ES* = -0.081). Still, for both reference attributes most responses were neutral.

The least biased responses were observed for the publication channel, that is, the respondents did not exhibit preferences for either journal or conference proceedings publications.

### Pairwise comparisons of clues

**Fig. 2:**
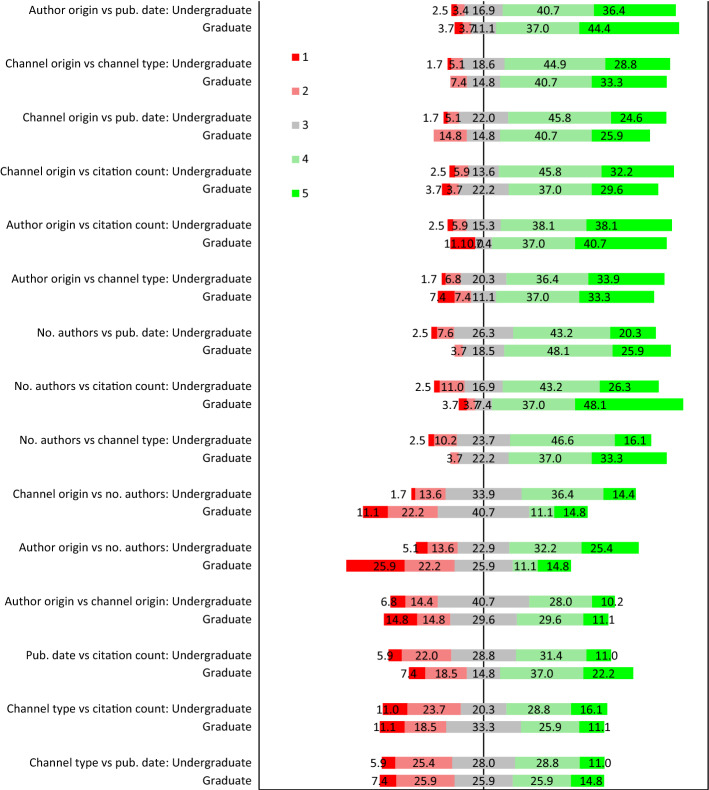
A diverging stacked bar graph showing the percentage distribution of respondents’ perceived trust in the 15 pairs of six publication reference attributes on a 5-item Likert scale

Figure 2 shows a summary of the respondents’ trust in publication reference attributes when presented with all pairwise combinations. Again, respondents exhibited a preference for publication date and number of citations in most pairs involving these attributes. The only exception to this was the contrasting of publication date and number of citations to publication channel type. For both pairs the responses did not reveal a bias. When contrasting publication date and number of citations, the responses were relatively balanced although there was somewhat a small bias in favour of citation counts.

Next, when contrasting name origin against publication channel type (journal vs. conference proceedings), most of the respondents expressed a preference for trusting publication channel type. Similarly, most respondents exhibited a preference for trusting the publication channel over the number of authors. No biases were observed for the remaining pairs, namely, author name origin versus number of authors, publication origin versus number of authors, and author origin versus publication origin.

Three pairs revealed significant differences between graduates and undergraduates. Undergraduates exhibited a stronger preference for trust in the number of authors compared to the more balanced graduate responses (*W* = 1157.5, *p* = 0.021, *ES* = 0.273). Undergraduates also preferred to trust the number of authors over the origin of the authors, whereas graduates were more impartial (*W* = 965.5, *p* = 0.001, *ES* = 0.389). Although both undergraduates and graduates exhibited a preference for number of citations over number of authors, the preference for number of citations was stronger among the graduate students (*W* = 2010.5, *p* = 0.025, *ES* = 0.262).

### Attitudes towards research

**Fig. 3:**
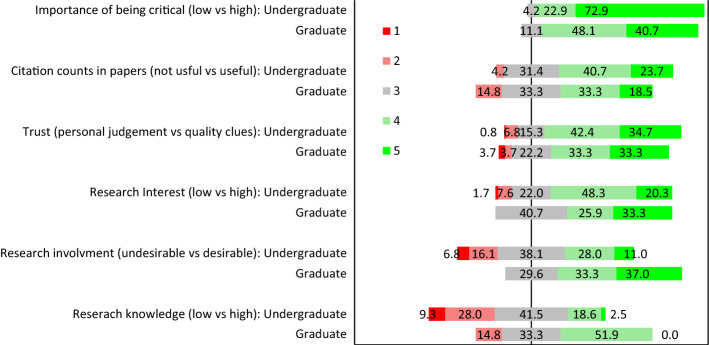
A diverging stacked bar graph showing the baseline percentage distribution of respondents’ self-reported knowledge about research, attitude to research, and experience with research on a 5-item Likert scale

When asked to score their trust in their own judgement of the research claim versus relying on quality signs of the research publication, both graduates and undergraduates tended towards relying on quality signs of the reference rather than their own judgements of the actual claim (see Fig. 3). Also, a majority of the graduate and undergraduate students reported that it was important to be critical about research. However, the undergraduate students rated the importance of being critical significantly more important than the graduates (*W* = 1073.0, *p* = 0.001, *ES* = 0.326). Further, although opinions varied more among undergraduate students, a majority (68.3%) expressed a positive interest in research. No graduate students reported a lack of interest in research, but 40.7% gave a neutral response. Among the graduate students 92.6% reported that they would like to learn more about research, while 88.1% of the bachelor students expressed this aspiration. Figure 3 shows that most of the graduate students would like to be more involved in research activities, while the responses among the undergraduates were more varied. This difference was also significant (*W* = 2307.5, *p* < 0.001, *ES* = 0.449). Most respondents were also positive to a proposal of including citation counts in the in-text citation and/or reference list within a publication.

### Links to trust

The responses to the three sections of the questionnaires were also correlated using Spearman correlations. Relevant significant correlations included the following: The respondents’ self-reported knowledge of research correlated positively with their preference for publication channel type (*r*_*s*_(143) = 0.205, *p* = 0.013), and whether they found research interesting (*r*_*s*_(143) = 0.421, *p* < 0.001). Respondents’ agreement with the proposal of including citation counts in papers correlated with trust in citations (*r*_*s*_(143) = 0.286, *p* < 0.001), trust in publication date (*r*_*s*_(143) = 0.248, *p* = 0.002), and trust in number of authors (*r*_*s*_(143) = 0.233, *p* = 0.005). The responses to the question about how interesting research is correlated with their trust in clues exhibited by the publication reference (*r*_*s*_(143) = 0.222, *p* = 0.007), importance of being critical to research (*r*_*s*_(143) = 0.336, *p* < 0.001), and a wish to be more involved in research (*r*_*s*_(143) = 0.541, *p* < 0.001).

### Additional comments

The free-text responses to the questionnaire revealed several insights. One respondent pointed out the problems associated with positively crediting citations resulted from criticism of flaws. Another respondent stated that the publication reference attributes just serve as simple heuristics and not fixed rules. One respondent pointed out that the quality of the article is more important than easily measurable attributes:

For me, personally, it doesn't necessarily revolve around where the research was done, or what the names of the scientists are. The deciding factors for me, is whether the article was written after good and thorough research, which I do believe is more likely if it is published in a well-known and highly regarded journal, rather than one that is not.

A similar comment regarding research quality was made by another respondent but with a diverging view concerning prestigious journals:

My main method of assessing papers is based on the merits of their methodology and statistics. After the replication crisis I have a lot of distrust towards most journals, and it has been shown that highly prestigious journals are not more likely to have their papers replicated than less prestigious journals.

The respondent argued for critical reading and “in general we should act as peer review ourselves whenever we read a paper.” The same respondent shared the following strategy for relating to diverging claims:

If two researchers claim two different results, one negative and one positive then the negative is more likely because if the studies have sufficient power they should both detect the results, if they don't it seems to be more likely to be a false positive. Furthermore, researchers are more likely to want positive results for their own hypothesis, so this bias is also a factor. In the cases when they claim effects in two different directions, I can't decide between them and would rather assume that there is no effect at all.

Another respondent also pointed to the importance of the text content in that a trust in claims should be formed based on the quality of the paper, the method that is used, the size of the experiment as well as how the results corresponded with the results of related research.

One respondent reflected over the difficulty of being critical of research claims. On one side untrained readers should not be gullible to arbitrary claims, yet they need to acknowledge that a researcher, typically also a domain expert, knows more about a particular topic than themselves. The respondent exemplified the social media trend “do your own research” as problematic in cases where juvenile activists achieve more support for their claims than claims stemming from thorough research conducted by domain experts.

One respondent expressed that it would be useful in this context with a ranking of the trustworthiness of the organisations or institutions that publish the papers. Another respondent indicated a need for information about how renowned the respective publication channel was.

## Discussion

One may assume that a trained researcher with specific domain expertise is more likely to assess claims in the literature based on their actual substance rather than attributes of the citing sources. Yet, the observation that most of the student respondents were more inclined to trust their impressions of reference quality rather than their own judgement implies that it is relevant to study how reference information is perceived. It is indeed problematic if untrained readers' impressions of scientific claims are biased by reference information without logical connections to the claims. Knowledge about how reference information causes bias among untrained readers may contribute to improving research literacy training. It is a paradox that readers without the training to assess quality signs of references may be more dependent on these clues than trained researchers. Although trained researchers are more likely to be able to correctly assess quality signs of references, they are probably less inclined to blindly rely on such clues.

### Citation counts

The results give support to hypothesis H1, that is, untrained readers perceive references to papers with many citations more trustworthy than papers with few citations. This result is aligned with the large body of literature on citation-based research assessments (Lindsey, 1989; Onodera & Yoshikane, 2015; Phelan, 1999). No explanation concerning citations was given in the questionnaire. This result indicates that most of the respondents have some notion of what citations are. This also seems consistent with the findings of Strømsø et al., (2013) who found that students consider second order sources cited in documents when reading about controversial topics. One would expect graduate students to be more aware of citations, perhaps for their more research-intensive curriculum and research-active teachers. However, the results do not indicate that undergraduates have less awareness of the importance of citations. It is also possible that the undergraduate students have been introduced to the citation regime during their studies. Citations were discussed in all the classes where the questionnaire was deployed, but steps were taken to deploy the questionnaire before these discussions took place so as to capture the students’ perceptions without influence from the course contents. Another explanation may be that several social media platforms utilise mechanisms that are similar to citations such as the number of likes, number of views, and hashtag references. The free text comments also indicate that some of the students seem to have a certain understanding of the benefits and limitations of citation statistics.

The inclusion of citation information in this study was hypothetic. Fortunately, citation information is usually not a part of the reference information within a citing text in practice, and a reader is thus unlikely to be aware of the citation counts and consequently unlikely to be biased by such information. A reader typically will need to take explicit action to identify citation information in some database. If a reader knows that it is possible to look up citation information and knows how to do so, one may hope that the reader also has some fundamental knowledge about citations, including their limitations. However, such insights cannot be assumed.

On the other hand, citation information appears to become increasingly pervasive on the web. For example, many digital libraries will list citation information on the landing pages of DOI links which may attract readers’ attention and cause bias. It is thus important to be aware that untrained readers could be tempted to trust citations when resolving conflicts.

### Publication date

The observations give support to hypothesis H2, that is, untrained readers perceive references to recently published work more trustworthy than less recently published work. Again, this result is as one would expect and aligns with the literature (Glänzel & Schoepflin, 1995; Hörlesberger et al., 2013; Klavans & Boyack, 2008). It seems intuitive that more recently published work is more relevant and aligns with the current situation than less recently published work, and perhaps that newer results improve upon and replace previous ones. However, publication date alone cannot be used as a quality indicator as a recent low-quality or small-scale study should not automatically replace a thorough and well-designed large-scale study conducted decades ago. In the context of technology a more recent study is more likely to represent the current context, but this may not be true if the research is outdated. Clearly, older work cannot represent technological paradigms that emerged after the work was published. The technology students in this study were not asked to assess the contents, while the non-technology students in Bråten et al. (2011) placed more emphasis on content than date of publication. Thus, the support for H2 is constrained by the assumption that the content is not taken into consideration. Moreover, there is likely an effect of discipline as technology students may be more conscious of the time factor and rapid technological paradigm shifts than students in other disciplines such as history where paradigm shifts occur less frequent.

Liu (2021) raised the issue of ambiguous publication dates as journal articles increasingly are published online before they appear in print. It is not uncommon for a publication to be available online for several years before appearing in print. The final publication is usually assigned one of these publication dates which may lead to uncertainty regarding when the work was first shared with the public.

The three questions contrasting new and old publications contained pairs that varied noticeably in time by several decades. The questionnaire did not explore the effect of publication date when these differ only by a couple of years.

It is probably unwise to solely rely on publication dates as this information should be viewed in context of other information. Another consequence of the potential bias associated with publication dates is their presence in in-text citations. A traditional citation and corresponding reference are devices to help researchers locate the source to follow up aspects of a text in detail. Author-year citation formats such as those declared by APA give the reader clues to who the authors are and how recent the work is as they read the text without having to consult the reference list. However, this could be a potential cause for bias. The author-year format is also most probably serving as a stronger mnemonic when going back and forth between the text and the reference list. Though, the need for mnemonic aids may be less of an issue with electronic papers where the reference connected is accessible via a single click on a hyperlink. Number format citations such as those used in IEEE and ACM publications (and sometimes ISO 690) are purely functional as compact reference markers revealing no clues to details about the publication and may thus pose a smaller risk of bias. In any case, the results support the training of readers to be vigilant regarding publication dates.

### Number of authors

The results also give support to hypothesis H3, that is, untrained readers perceive references with multiple authors more trustworthy than single authored references. Again, this agrees with the literature documenting connections between the number of authors and degree impact (Bartneck & Hu, 2010; Bu et al., 2018a, 2018b; Larivière et al., 2015; Melin & Persson, 1996; Sandnes, 2022; Wuchty et al., 2007; Xu et al., 2015). It is interesting that the degree of trust in the number of authors differed significantly between undergraduates and graduates. One could argue that graduate students with an assumed deeper knowledge of research would be more aligned with what is reported in the literature. The opposite was in fact observed, that is, undergraduates’ perceptions were more aligned with the findings in the research literature. As both undergraduates and graduate students major in similar subject areas (technology), one may speculate that this trust difference in single versus multiple authors likely reflects the varied experience between the undergraduate and graduate students. It seems possible that undergraduates with intrinsic ideology valuing collaboration would perceive multiple-authored research as more trustworthy, compared to graduates who typically work less in teams and have more experience reading research publications. It is also important to note that fewer graduate students participated in this study than undergraduate students.

The fact that the untrained readers in this study seemed influenced by the number of authors gives some reason for concern, even though many studies have observed positive correlations between the number of authors and positive qualities. The arguments for the benefits of multiple authors rest on an assumption that all the authors are active contributors to the work. However, honorary authorship is still a common problem (Greenland & Fontanarosa, 2012), whereupon individuals are listed as authors without having contributed to the work. Readers’ trust in author lists may be higher for publication channels that implement mandatory author contribution declarations, yet such declarations are based on trust and could be untruthful. In conclusion, readers should be vigilant when considering the number of authors, although the potential bias caused by the number of authors seems to pose a lower risk than citation counts and publication dates.

### Geo-economic bias

The results related to geographical origin of the authors and publication channel in terms of name show a small preference for research published by authors from high GDP-per-capita countries and published in publication channels hosted in high GDP-per-capita countries, thus giving weak support to hypothesis H4 and H5. That is, untrained readers perceive references by authors with names often associated with high GDP-per-capita countries as more trustworthy than those associated with low GDP-per-capita countries, and untrained readers perceive references to papers published in publication channels hosted in high GDP-per capita countries more trustworthy than those hosted in low GDP-per-capita countries.

One may speculate whether this bias is due to prejudice towards low GDP-per-capita countries or whether it is connected to an impression that quality is related to GDP-per-capita. It is also interesting to observe that the expressed scepticism towards research associated with low GDP-per-capita countries is stronger among the graduate students who also included international students from several low GDP-per-capita countries. It is indeed a matter of concern if readers show favouritism towards certain claims based on the names of the authors or the publication channel.

Although not addressed herein, one could expect there to be a certain level of prejudice towards certain countries among the general population and trained researchers. Still, we would expect that most trained researchers are impartial to the geographical origins of authors’ names as one can no longer reliably infer the country of residence for an individual from their name alone. This also agrees with the findings of Long et al. (2009) where author origin had no effect.

However, if also taking the authors’ affiliation (institution and/or country) into consideration, one would probably expect to observe a noticeable bias also among researchers as certain countries and institutions have different profiles and reputations. In fact, Long and colleagues (2009) argued that it matters where the authors received their education.

Researchers’ preference for international publication channels over national publication channels are well documented (Li et al., 2018; Meneghini et al., 2006; Nazarovets, 2020; Rabinovich, 1992). However, seemingly less effort has been devoted to relating trust to a country’s GDP-per-capita. Trained researchers are most likely familiar with the relevant journals of their field irrespective of where these are hosted. They may also vet an unknown source using indexing databases and journal rankings. They are thus probably likely to favour high GDP-per-capita publication channels in situations when they encounter new or unfamiliar publication channels. Such scepticisms towards unknown sources could be considered valid and even healthy. For an untrained reader, one could argue that the country’s GDP-per-capita could serve as a reasonable quality heuristic to rank the quality of journals bearing that country’s name. On the contrary, there is nothing stopping anyone in a low GDP-per-capita country to inaugurate a journal with a title that includes the name of a high GDP-per-capita country or with the label “international” to influence readers’ and authors’ trust. Also, GDP-per-capita is a parameter that varies over time in patterns totally unrelated to the content of a specific journal.

This study addressed the perceived geographical affiliation of the authors and journals. Perception of a geographical affiliation is also affected by the geographical affiliation of the reader as preferences indeed differ across cultures (Jian et al., 2010a, 2010b, 2010c). A reader will have a deeper understanding of the research stemming from their own country and perhaps have a more incomplete and shallow understanding of certain other countries and their research traditions. Although the respondents at graduate level had varying cultural affiliations, the sample was too small to make reliable inferences across cultural boundaries. One possible avenue of follow-up research may be to explore how perceptions of trustworthiness in research differ across cultures.

### Publication channel type

The results do not give support to hypothesis H6, that is, untrained readers perceive references to journal papers more trustworthy than papers in conference proceedings. This is quite contrary to common attitudes among trained researchers where journals have a higher status than conference proceedings (Franceschet, 2010; Kim, 2019; Lee, 2019; Mannocci et al., 2019). The level of training is one probable factor that can explain this observation as most of the respondents did rate their own research knowledge as medium to low. An untrained reader may be more open to claims irrespective of where they are published since they may not have been introduced to the research system and the traditions of journals and conferences. A trained researcher in certain disciplines, however, will have been indoctrinated to follow the tradition of attempting to achieve prestige by aiming for the most prestigious journals and publish early work-in-progress in conference proceedings. Regardless, trained researchers may be more driven by specific knowledge about the prestige and perceived quality of specific publication channels than the category itself. This is especially the case in disciplines where the distinction between conference and journal publications is becoming less important (Sandnes, 2021a). However, as evidenced by the results, it is hard for untrained readers to assess the quality of the publication channel simply based on its name. This could be a potential threat to the public discussion if favour is uncritically placed on the lower of two sources with diverging claims where the two sources are at the different end of the quality scale. Fortunately, the results indicate that the risk of publication channel bias is small.

As previous studies have observed differences between how researchers with domain knowledge versus laypersons assess a research publication (Bråten et al., 2011; von der Mühlen et al., 2016), it is also possible that the results reported for the technology students herein would have been different with a cohort of students from a different field of study. One may interpret the students’ ignorance or impartiality to the traditional caste hierarchy of journal publications over conference proceedings as a positive sign. This is especially true if such traditions and values are counterproductive with adverse consequences. Individual researchers may be slow to change their own practices and attitudes, while such changes may emerge with new generations of researchers. When comparing the results herein to the results of Bråten et al. (2011), there appears to be a pattern where students generally are aware of differences between textbooks, official reports, and newspapers, while being less knowledgeable about differences between scientific conference proceedings and journals.

### Implications

It is possible that the biases observed could be counteracted by many of the educational remedies proposed in the literature (Barzilai & Chinn, 2020), such as training lateral reading strategies (Breakstone et al., 2018; Wineburg & McGrew, 2019), discussing disagreements (Broomell & Kane, 2017), and collective sensemaking (Feinstein & Waddington, 2020). For example, to lessen the students’ observed misplaced trust in citations educators involving students in research-related coursework could set aside time to discuss the concrete role of citations with students, including their uses and misuses. Simply relying on the students’ preconception of what citations are is probably not enough. The students’ focus on relying on citations as a quality indicator could be shifted towards practices where the students actively follow citations and explore the contents of the citing sources thereby uncovering other related and more recent works that build on a given source.

Bachelor and master students should be introduced to the research system even though they are not going to pursue a research career. This will prepare the students to become what Feinstein et al. (2013) called *competent outsiders* capable of finding and interpreting the research they need in response to challenges they encounter.

For PhD students that explicitly train for a research career some of the literature points towards learning experiences that prepare students to be active participants in the research community, see for instance (Li, 2007; McGinn & Roth, 1999).

## Limitations

The observed biases were intentionally amplified by the study design as the respondents were asked to rely on the information in the references and ignore the substance of the claims (content). It must therefore be noted that the results do not provide evidence that respondents automatically are biased by references. What the results do indicate is untrained readers’ potential of being biased under given circumstances.

One challenge with this type of study is to balance the length of the questionnaire and the number of issues addressed. One respondent commented that the questionnaire was long. The number of responses was relatively limited but sufficient to elicit indications of general trends.

There is also a balance between minimising the influence on the responses versus giving clear instructions with the risk of influencing the responses. One respondent commented that it was unclear what the intention was when asked to choose according to the name of the author. This respondent deduced that it could refer to how renowned or famous the researcher is, the researchers’ gender, or (correctly) the origin of the author. The intention was purposely not mentioned so as not to lead the respondents towards a particular answer. The names used in the study were fictitious and did not correspond to any notable persons. Moreover, only family names (surnames) were given thereby not revealing any cues to the gender of the authors.

Another challenge with such a study is that the respondents need some basic insight to the context of the study, and hence the recruitment of students seems the most pragmatic option. The voluntary nature of the study may have affected the results as one would expect that students interested in research are more likely to accept the invitation to respond. Thus, we could expect that the respondents are somewhat biased towards the upper tier of the respective student populations.

One respondent pointed out that it was challenging to respond merely based on the claims and a set of references in the first part of the questionnaire. For some questions this respondent responded according to his or her own assessment of the actual claim, and for other questions the responses were based on the overall impression of the references and the claims. Several respondents may have adopted similar strategies, and this could have somewhat affected the results. However, the fact that each attribute was measured with three different questions may have helped reduce the effect of the actual claim on the results.

The results of this study echo students’ perceptions. It could also be relevant to target a similar study towards researchers (experts) to observe to what degree they are biased by publication information. Moreover, it may be relevant to target journalists and communication workers as they do not usually have explicit research training yet occasionally have to use research publications as a source.

## Conclusion

This study probed how readers without formal research training perceive trustworthiness of research based on publication reference attributes. The results showed that the number of citations and publication dates were the most influential attributes. Small biases for publications with many authors and against publication published in low GDP-per-capita journals were also observed. These observations are aligned with the literature and what one may expect to observe among trained researchers. A small preference for researchers associated with names originating from high GDP-per-capita countries was noted. There are no logical arguments to support such preferences or biases, and the respondents’ trust in authors with names originating in high GDP-per-capita countries is probably misplaced. The respondents did not exhibit any preference with regard to journals as opposed to conference proceedings while a trained researcher probably would place more trust in journals. One implication of these results is that teachers should set aside time to discuss the research system with students. In particular, students should be introduced to the limitations and possible misuses of citations.
